# Gastrointestinal Mucormycosis of the Jejunum in an Immunocompetent Patient: A Case Report

**DOI:** 10.1097/MD.0000000000006360

**Published:** 2017-04-21

**Authors:** Mengqing Sun, Xianming Hou, Xiaoting Wang, Ge Chen, Yupei Zhao

**Affiliations:** a Department of Surgery; b Department of General Surgery; c Department of Intensive Care Unit, Peking Union Medical College Hospital, Chinese Academy of Medical Sciences and Peking Union Medical College, Beijing, China.

**Keywords:** gastrointestinal bleeding, gastrointestinal tract, mucormycosis

## Abstract

**Rationale:**

Gastrointestinal Mucormycosis (GIM) is a kind of opportunistic fungal infection with poor prognosis. It usually occurs in patients with immune deficiency. We reported a case of immunocompetent male patient.

**Patient concerns:**

This patient was presented as abdominal distension and gastrointestinal bleeding.

**Diagnoses:**

A variety of hemostatic methods was ineffective to stop the bleeding. The patient finally received laparotomy, and the jejunum lesions were found.

**Interventions:**

Pathological examination confirmed it to be gastrointestinal mucormycosis in jejunum.

**Outcomes:**

However, after systemic anti-fungi therapy, the patient died of septic shock.

**Lessons:**

The diagnosis mainly relies on pathological examination. Early diagnosis and early application of systemic amphotericin B liposome were fundamental for improving the prognosis.

## 1 Background

Mucormycosis is an opportunistic infection caused by conditional fungal pathogens. It is characterized by vascular invasion by the hyphae, leading to thrombosis and necrosis. Mucormycosis can occur in the head and neck, lung, skin, gastrointestinal tract, and central nervous system, causing the corresponding symptoms. Most patients with gastrointestinal mucormycosis (GIM) are immunocompromised, and an immunocompetent host with GIM is rare. Here, we report a case of mucormycosis that affected the gastrointestinal tract. The patient had no evidence of immunosuppression. A literature review is also presented. Informed consent was obtained in this study. The Ethical Committee of Peking Union Medical College Hospital approved the present study, and the patient approved with a written consent before the surgery.

## 2 Case report

A 66-year-old male patient presented with a past medical history of rheumatic heart disease and mitral stenosis for more than 10 years and has been taking oral warfarin anticoagulation. Sixteen days before admission, he had abdominal distension, nausea, and fever, with a *T*_max_ of 39°C. Thirteen days earlier, the abdominal distention had become worse. An enema was performed, and the antibiotics moxifloxacin and levofloxacin were administered successively. Six days before admission, the patient developed difficulties eating, and received parenteral nutrition. On the morning of admission, the patient had dyspnea, with a respiratory rate (RR) of 30/min, and he came to the emergency room of our hospital.

### 2.1 Hospital course

**Figure 1:**
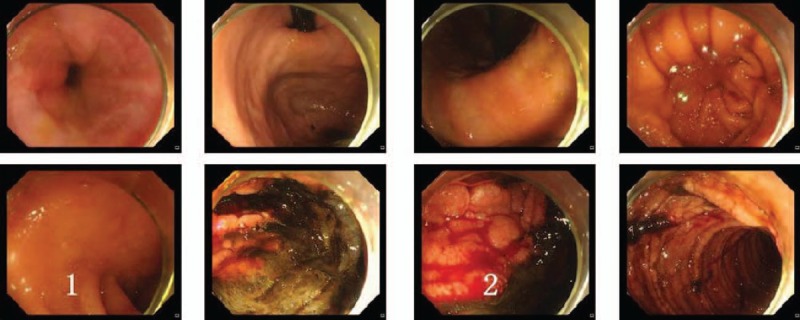
Gastroscopic examination. 1 = Multiple lesions were found in the horizontal portion of the duodenum, 2 = a small amount of fresh bleeding.

**Figure 2:**
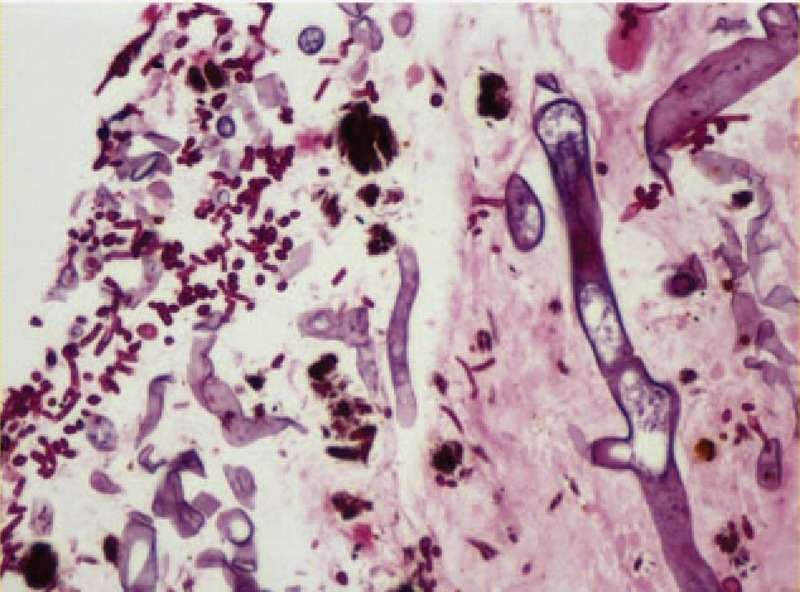
The pathological examination of the gastroscopic biopsy specimen. Fungal hyphae can be observed in the inflammatory exudates.

The patient's vital signs were as follows: *T*: 36.8°C, heart rate (HR): 156 bpm, RR: 28/min, blood pressure (BP): 91/63 mm Hg, and SpO_2_: 99%. The breathing sound of the bilateral lower pulmonary field was low, and scattered wet rales could be heard. The laboratory examination results were as follows: hemoglobin (HGB) 84 g/L, white blood cell (WBC) count 8.51×10^9^/L, neutrophil% (NEUT%) 83.2%, and potassium (K) 3.1 mmol/L; other results, including the complete blood count, liver function, renal function, and electrolytes, were normal. The patient was admitted to the intensive care unit (ICU) ward, and cefoperazone/sulbactam was prescribed. The patient had symptoms of gastrointestinal bleeding (GIB) during the night with hematochezia and positive occult blood in both his stool and gastric juice. The HGB was reduced to 65 g/L. Three days after admission, the patient received an examination by gastroscopy, and a jejunum nutrition tube was inserted. The gastroscopic results showed multiple lesions in the horizontal part of the duodenum, and a biopsy was performed (Fig. 1). The pathological examination suggested inflammatory exudates, and acute and chronic inflammation of the small intestinal mucosa. Fungal hyphae were seen in the inflammatory exudates, which were considered mucor (Fig. 2).

Due to the continuous gastrointestinal bleeding, an interventional therapy of artery embolization was performed on day 5 to stop the bleeding. The gastroduodenal artery proximal to the right gastroepiploic artery was embolized. The patient still had hematochezia after the intervention. A positron emission tomography-computer tomography (PET-CT) and an abdominal computer tomography angiography (CTA) examination were performed, but no obvious bleeding site was found.

Because of the ineffectiveness of the variety of hemostatic methods, laparotomy and intraoperative gastroscopy exploration were performed on day 6. During the operation, we observed adhesions of the jejunal wall 15 cm from the Treitz ligament with the surrounding small mesentery. There was a hard, palpable mass 5 cm in size. After decomposition of the adhesion, the site of chronic perforation could be observed in the jejunum wall. The site of the GIB was the proximal jejunum. The perforation site and the distended proximal jejunum were resected. A drainage tube was placed near the anastomosis, and a jejunum fistula tube was placed 50 cm from the anastomosis. The abdominal drainage fluid was collected for a pathogen culture.

The results of the culture returned on postoperation day (POD) 4 suggested *Candida albicans*, *Klebsiella pneumoniae*, and *Acinetobacter baumannii*. Caspofungin was prescribed for antifungal therapy. Cefoperazone/sulbactam, minocycline, and vancomycin were used for empirical antibiotic therapy. The patient's temperature returned to normal. Minocycline, vancomycin, and caspofungin were terminated on POD 11. The cefoperazone/sulbactam was withdrawn, and the abdominal drainage tube was removed on POD 14.

**Figure 3:**
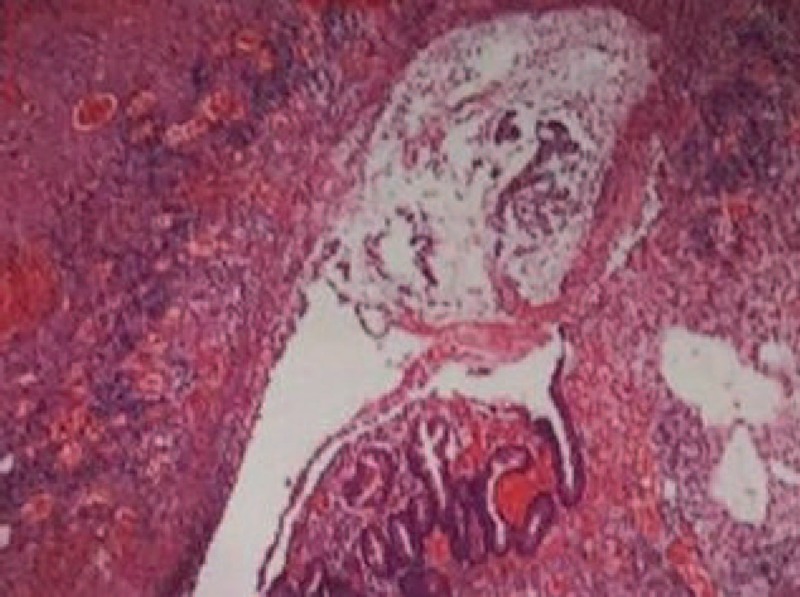
The pathological examination of the surgical specimen.

Heparin anticoagulant therapy was initiated on POD 17 for the rheumatic heart disease. Melena reappeared. The patient's HGB decreased to 86 g/L. Heparin was stopped, and the patient received a blood transfusion. The hematochezia did not improve. The plasma fungal D-glucan (G test) was 112.80 pg/mL. The pathologists re-examined the ulcer lesion of the surgical specimen and found degenerative fungal hyphae. The morphology and specific staining suggested it was a mucor infection (Fig. 3). Oral mycostatin and intravenous amphotericin B (Amp B) were initiated on POD 26. Amp B was initiated at a dose of 5 mg daily, which was gradually increased to 20 mg daily. The patient had a fever, abdominal distension, and an increase in bilirubin, with a total bilirubin (TBil) of 36.2 mol/L and a direct bilirubin (DBil) of 26.7 mol/L. We considered these results adverse effects of Amp B, and gradually reduced the dose to 15 mg/d. After 20 days of medication, we withdrew the Amp B and used the single drug mycostatin. During the drug treatment, the patient's gastrointestinal bleeding gradually stopped, and the temperature remained normal. Six weeks after the operation, the patient was transferred from the ICU to the general surgery ward to continue the treatment of oral mycostatin until drug withdrawal. The total course of antifungal treatment with Amp B and mycostatin was 4 weeks. Nine weeks after the operation, the jejunal fistula tube was removed. The patient's body temperature was normal, and there was no evidence of gastrointestinal bleeding. The patient was discharged.

Four days after discharge, the patient again developed nausea, vomiting, and left abdominal pain with a body temperature up to 38.9°C. The following day, the patient developed abdominal distension. After an enema, about 1000 mL of loose stool was discharged. Subsequently, the patient had diarrhea approximately 8 times a day, with a total of approximately 1000 mL. The patient complained that he was thirsty. One week after discharge, the patient had delirium, weak breathing, confusion, and oliguria. He was re-admitted to the hospital, and septic shock was considered. Anti-infection therapy and active drug treatment were ineffective, and the patient was declared dead.

## 3 Discussion

Mucormycosis can occur in many parts of the human body, such as the head and neck, lung, skin, gastrointestinal tract, central nervous system, and primarily in the nasal cavity.^[1,2]^ GIM accounts for only 7% of all cases, but the mortality rate can be as high as 85%.^[3]^ Mucormycosis in the gastrointestinal tract is most commonly observed in the stomach, followed by the small intestine and colon.^[4]^ The clinical presentation of the disease can be manifested as ischemic bowel disease or gastrointestinal bleeding. The diagnosis depends on the pathological examination.

The prognosis of GIM is poor, and the disease has a high mortality. Most patients have factors of immune deficiency, such as organ transplantation, hematological malignancies, and glucocorticoid steroid therapy. The pediatric literature has reported a number of cases of neonatal necrotizing enterocolitis caused by mucormycosis.^[5,6]^ GIM in an immunocompetent host is rare.

We searched the cases of GIM in immunocompetent adults that have been reported in the English literature since 2000. A total of 9 cases were retrieved. These cases are summarized in Table 1.

**Table 1:**
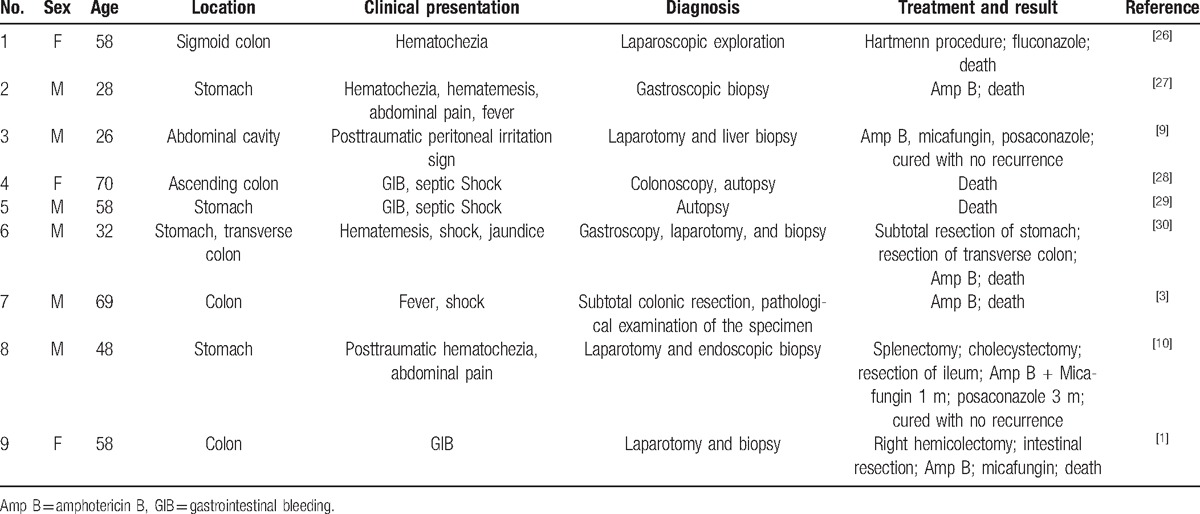
Summary of the cases of gastrointestinal mucormycosis in immunocompetent adults that are reported in the English language literature since 2000.

In the 9 cases, we found the following: 4 cases of GIM occurred in the stomach; 5 cases of GIM occurred in the colon (including 1 case occurring both in the stomach and the colon); and 1 case of GIM occurred in the abdominal cavity after a trauma. Here, we report an immunocompetent adult case of GIM occurring in the jejunum, which has not been reported in the literature. However, GIM of the jejunum has been reported in immunocompromised patients.^[7]^ Agha et al^[8]^ summarized 87 cases of GIM in immunodeficient patients, and only 1 case occurred in the jejunum. The diagnosis of this disease mainly relies on the pathological examination. All 9 cases shown in Table 1 were diagnosed by surgery, endoscopic biopsy, or autopsy. The diagnosis of the patient we reported here was also diagnosed by the pathology of the gastroscopic and surgical biopsy.

Almost all the cases reported in the literature had different underlying symptoms, such as fever, respiratory symptoms, or trauma, and then gradually developed gastrointestinal bleeding, such as hematochezia or hematemesis. Once gastrointestinal bleeding appeared, the patient's condition tended to deteriorate rapidly with the occurrence of septic shock or multiple organ failure. Of the 9 patients we summarized, only 2 patients were cured without recurrence.

Most of the mucormycosis cases are caused by environmental exposure. The most common site of infection is the nasal cavity. GIM in an immunocompetent host is very rare. It is likely that an underlying disease, such as respiratory infections or viral infections, causes a temporary decline in the immune system, and the fungi in the nasal cavity, parapharynx, or other common infection site invade the blood vessel walls and disseminate to the gastrointestinal tract, causing ulceration and perforation. In this case, the patient developed a fever 16 days before admission, which may have been the cause of the temporary decline in immunity. The rapid increase in GIB may be related to the long-term anticoagulation because of rheumatic heart disease and mitral stenosis.

The treatment of GIM includes surgical resection of the lesions, systemic application of antifungal drugs, and support therapy. For patients with an immunodeficiency, correction of the underlying disease is also very important.

### 3.1 Surgical resection of the lesion

The surgical treatment of GIM usually occurs in a passive time, such as during emergency laparotomy, debridement, and drainage. The symptoms arising before the surgery are usually nonspecific emergent presentations, such as bleeding and perforation. In addition, as reported in the present case, the diagnosis of the disease also depends on surgical resection or biopsy of the specimen, followed by a pathological examination rather than microbiological culture.^[1]^ Therefore, it is difficult to diagnose the disease before surgery.

Regarding the purpose of the surgery, because the disease can cause thrombosis and necrosis of the gastrointestinal tract and the surrounding tissue, the effectiveness of only antifungal therapy without surgery will be poor. There are 2 cases of successful treatment shown in Table 1.^[9,10]^ A study of the 2 cases suggested that in operating on such a special infectious disease, complete elimination of the necrotic tissue and sufficient drainage should be the purpose of the operation. In addition, it may be helpful for the patient.^[9–11]^

### 3.2 Systemic therapy

The systemic application of antifungal drugs is another major basis for the treatment of mucormycosis. The early initiation of antifungal therapy improves the outcome of mucormycosis infection.^[12]^ Unfortunately, the experience of using antifungal drugs reported in the literature is mostly for immunocompromised patients with no gastrointestinal involvement. However, based on experiences of antifungal therapy in other lesions, we can generally conclude the following.

Amphotericin B is currently recommended as a first-line drug. Amp B liposome is also noteworthy. Compared with deoxycholate and the lipid complex forms of amp B, amp B liposome has a relatively low renal toxicity, which is advantageous for long-term use and larger doses of medication. Moreover, some reports have noted that the application of amp B liposome is more effective for patients with mucormycosis.^[13,14]^

Fluconazole, voriconazole, and itraconazole do not have reliable activity against mucormycosis.^[15]^ Posaconazole is not used as a first-line drug because it is unsatisfactory in plasma concentrations,^[15,16]^ ineffective in animal models,^[17]^ and has failed in prophylactic use.^[18]^ However, it is noteworthy that posaconazole has demonstrated efficacy in the patients with polyenes intolerance, such as neutropenia.^[19]^ Therefore, it was a choice between combination therapy and remedial treatment.

Echinocandin drugs, such as caspofungin, are first-line antifungal drugs. They are mainly used in invasive *Aspergillus* infections in which other antifungal agents are ineffective or poorly tolerated. Although the echinocandins (eg, caspofungin) have no in vitro activity against the agents of mucormycosis,^[20]^*Rhizopus oryzae*, the most common cause of mucormycosis, expresses the target enzyme for echinocandins, suggesting that these agents may have clinical utility.^[21]^ For some types of mucor, several laboratory studies suggest that echinocandins combined with amp B can produce a better effect.^[22]^ However, this conclusion still lacks the support of large-scale clinical trials.

### 3.3 Other auxiliary therapy

#### 3.3.1 Iron-chelating agents

Iron is required by virtually all microbial pathogens for growth and virulence.^[23]^ Unlike deferoxamine combined with iron that can be used as an iron carrier by pathogens and enhance the pathogenicity, after combining with iron, deferasirox can reduce the iron content to reduce the fungal load and, thus, improve survival. Therefore, deferasirox has a potential therapeutic value in treating mucormycosis. However, in some clinical studies, the combination of deferasirox and amp B did not produce a better prognosis.^[24]^ Whether deferasirox can improve the prognosis or be used as an alternative treatment option still requires confirmation with more reliable clinical studies. In contrast to patients with diabetes or ketoacidosis, our immunocompetent patient had normal iron metabolism, and the effectiveness of deferasirox remained questionable.

#### 3.3.2 Proinflammatory cytokines, such as interferon–γ, and granulocyte-macrophage colony-stimulating factor (GM-CSF)

These drugs are used to correct the patients’ immune condition, thereby improving their prognosis. The effectiveness of such treatments is still controversial. For immunocompetent patients with GIM, no theoretical basis exists for this application.

From the literature, we find that in the 9 cases reported, only 2 patients were successfully cured with no relapse, and both cases involved posttraumatic (gunshot wounds, car accident) onsets. One patient underwent multiple debridement and drainage, and the other had a splenectomy, subtotal colectomy, terminal ileum resection, and multiple intraperitoneal irrigations.^[9,10]^ The systemic antifungi therapy in both of these patients included amp B, micafungin, and posaconazole. The basic drug regimen is amp B, combined with micafungin, as soon as possible after the diagnosis. Depending on the patient's general condition, such as leukopenia, posaconazole could be used. More interestingly, in a report of invasive mold infections after combat-related injuries, of a total of 37 patients, 16 were diagnosed as with mucormycosis, of which only 3 patients died, although the majority of patients had limb lesions.^[25]^ It appears that mucormycosis caused by trauma has a relatively good prognosis.

## 4 Conclusions

Gastrointestinal mucormycosis in immunocompetent patients is a rare disease, especially in the jejunum. The disease has a high mortality. The diagnosis of this disease mainly relies on a pathological examination, either by surgery, endoscopic biopsy, or autopsy. For immunocompetent patients with GIM, early diagnosis and early application of systemic amp B liposome were fundamental for improving the prognosis. Aggressive surgical treatment is also important. Patients with a traumatic onset appear to have a relatively good prognosis.

## References

[1] Antony SJ Parikh MS Ramirez R Gastrointestinal mucormycosis resulting in a catastrophic outcome in an immunocompetent patient. Infect Dis Rep 2015;7:6031. 26500741 10.4081/idr.2015.6031 PMC4593887

[2] Petrikkos G Skiada A Lortholary O Epidemiology and clinical manifestations of mucormycosis. Clin Infect Dis 2012;54 Suppl 1:S23–34. 22247442 10.1093/cid/cir866

[3] Choi HL Shin YM Lee KM Bowel infarction due to intestinal mucormycosis in an immunocompetent patient. J Korean Surg Soc 2012;83:325–9. 23166893 10.4174/jkss.2012.83.5.325 PMC3491236

[4] Spellberg B Gastrointestinal mucormycosis: an evolving disease. Gastroenterol Hepatol 2012;8:140–2. PMC3317515 22485085

[5] Lewis RE Kontoyiannis DP Epidemiology and treatment of mucormycosis. Future Microbiol 2013;8:1163–75. 24020743 10.2217/fmb.13.78

[6] Vallabhaneni S Walker TA Lockhart SR Centers for Disease Control and Prevention. Notes from the field: fatal gastrointestinal mucormycosis in a premature infant associated with a contaminated dietary supplement: Connecticut, 2014. MMWR Morb Mortal Wkly Rep 2015;64:155–6. 25695322 PMC4584706

[7] Martinello M Nelson A Bignold L We are what we eat!” Invasive intestinal mucormycosis: a case report and review of the literature. Med Mycol Case Rep 2012;1:52–5. 24371738 10.1016/j.mmcr.2012.07.003 PMC3855875

[8] Agha FP Lee HH Boland CR Mucormycoma of the colon: early diagnosis and successful management. AJR Am J Roentgenol 1985;145:739–41. 3875993 10.2214/ajr.145.4.739

[9] Van Sickels N Hoffman J Stuke L Survival of a patient with trauma-induced mucormycosis using an aggressive surgical and medical approach. J Trauma 2011;70:507–9. 21307754 10.1097/TA.0b013e31820784ff

[10] Machicado JD Younes M Wolf DS A rare cause of gastrointestinal bleeding in the intensive care unit. Healthcare-associated mucormycosis. Gastroenterology 2014;146: 911, 1136-1137. 10.1053/j.gastro.2013.11.038 24560854

[11] Roden MM Zaoutis TE Buchanan WL Epidemiology and outcome of zygomycosis: a review of 929 reported cases. Clin Infect Dis 2005;41:634–53. 16080086 10.1086/432579

[12] Chamilos G Lewis RE Kontoyiannis DP Delaying amphotericin B-based frontline therapy significantly increases mortality among patients with hematologic malignancy who have zygomycosis. Clin Infect Dis 2008;47:503–9. 18611163 10.1086/590004

[13] Gleissner B Schilling A Anagnostopolous I Improved outcome of zygomycosis in patients with hematological diseases? Leuk Lymph 2004;45:1351–60. 10.1080/10428190310001653691 15359632

[14] Ruping MJ Heinz WJ Kindo AJ Forty-one recent cases of invasive zygomycosis from a global clinical registry. J Antimicrob Chemother 2010;65:296–302. 20008047 10.1093/jac/dkp430

[15] Sun QN Fothergill AW McCarthy DI In vitro activities of posaconazole, itraconazole, voriconazole, amphotericin B, and fluconazole against 37 clinical isolates of zygomycetes. Antimicrob Agents Chemother 2002;46:1581–2. 11959605 10.1128/AAC.46.5.1581-1582.2002 PMC127128

[16] Ullmann AJ Cornely OA Burchardt A Pharmacokinetics, safety, and efficacy of posaconazole in patients with persistent febrile neutropenia or refractory invasive fungal infection. Antimicrob Agents Chemother 2006;50:658–66. 16436724 10.1128/AAC.50.2.658-666.2006 PMC1366875

[17] Ibrahim AS Gebremariam T Schwartz JA Posaconazole mono- or combination therapy for treatment of murine zygomycosis. Antimicrob Agents Chemother 2009;53:772–5. 18936190 10.1128/AAC.01124-08 PMC2630615

[18] Lekakis LJ Lawson A Prante J Fatal rhizopus pneumonia in allogeneic stem cell transplant patients despite posaconazole prophylaxis: two cases and review of the literature. Biol Blood Marrow Transplant 2009;15:991–5. 19589489 10.1016/j.bbmt.2009.04.007

[19] van Burik JA Hare RS Solomon HF Posaconazole is effective as salvage therapy in zygomycosis: a retrospective summary of 91 cases. Clin Infect Dis 2006;42:e61–5. 16511748 10.1086/500212

[20] Pfaller MA Marco F Messer SA In vitro activity of two echinocandin derivatives, LY303366 and MK-0991 (L-743,792), against clinical isolates of *Aspergillus*, *Fusarium*, *Rhizopus*, and other filamentous fungi. Diagn Microbiol Infect Dis 1998;30:251–5. 9582584 10.1016/s0732-8893(97)00246-0

[21] Ibrahim AS Bowman JC Avanessian V Caspofungin inhibits *Rhizopus oryzae* 1,3-beta-D-glucan synthase, lowers burden in brain measured by quantitative PCR, and improves survival at a low but not a high dose during murine disseminated zygomycosis. Antimicrob Agents Chemother 2005;49:721–7. 15673756 10.1128/AAC.49.2.721-727.2005 PMC547300

[22] Reed C Bryant R Ibrahim AS Combination polyene-caspofungin treatment of rhino-orbital-cerebral mucormycosis. Clin Infect Dis 2008;47:364–71. 18558882 10.1086/589857 PMC2793535

[23] Bullen JJ Rogers HJ Spalding PB Natural resistance, iron and infection: a challenge for clinical medicine. J Med Microbiol 2006;55 (Pt 3):251–8. 16476787 10.1099/jmm.0.46386-0

[24] Spellberg B Ibrahim AS Chin-Hong PV The Deferasirox-AmBisome Therapy for Mucormycosis (DEFEAT Mucor) study: a randomized, double-blinded, placebo-controlled trial. J Antimicrob Chemother 2012;67:715–22. 21937481 10.1093/jac/dkr375 PMC3383100

[25] Warkentien T Rodriguez C Lloyd B Invasive mold infections following combat-related injuries. Clin Infect Dis 2012;55:1441–9. 23042971 10.1093/cid/cis749 PMC3657499

[26] Sakorafas GH Tsolakides G Grigoriades K Colonic mucormycosis: an exceptionally rare cause of massive lower gastrointestinal bleeding. Digestive and liver disease: official journal of the Italian Society of Gastroenterology and the Italian Association for the Study of the Liver 2006;38:616–7. 10.1016/j.dld.2006.03.018 16750662

[27] Shiva Prasad BN Shenoy A Nataraj KS Primary gastrointestinal mucormycosis in an immunocompetent person. J Postgrad Med 2008;54:211–3. 18626171 10.4103/0022-3859.41805

[28] Anand J Ghazala K Chong VH Massive lower gastrointestinal bleeding secondary to colonic mucormycosis. Med J Malaysia 2011;66:266–7. 22111457

[29] Ryan O Frohlich S Crotty TB Rhizopus microsporus infection in an immunocompetent host: a case of immunoparalysis? Anaesth Intensive Care 2012;40:367–8. 22417051

[30] Lalwani S Govindasamy M Gupta M Gastrointestinal mucormycosis–four cases with different risk factors, involving different anatomical sites. Indian journal of gastroenterology: official journal of the Indian Society of Gastroenterology 2012;31:139–43. 22744237 10.1007/s12664-012-0215-z

